# Completeness and timeliness of *Salmonella *notifications in Ireland in 2008: a cross sectional study

**DOI:** 10.1186/1471-2458-10-568

**Published:** 2010-09-22

**Authors:** Nathalie Nicolay, Patricia Garvey, Niall Delappe, Martin Cormican, Paul McKeown

**Affiliations:** 1 European Programme for Intervention Epidemiology Training (EPIET), European Centre for Disease Prevention and Control (ECDC), Stockholm, Sweden; 2 Health Protection Surveillance Centre, 25-27 Middle Gardiner Street, Dublin 1, Ireland; 3 National Salmonella Reference Laboratory, Bacteriology Department, Galway University Hospital, Ireland

## Abstract

**Background:**

In Ireland, salmonellosis is the second most common cause of bacterial gastroenteritis. A new electronic system for reporting (Computerised Infectious Disease Reporting - CIDR) of *Salmonella *cases was established in 2004. It collates clinical (and/or laboratory) data on confirmed and probable *Salmonella *cases. The authors studied the completeness and the timeliness of *Salmonella *notifications in 2008.

**Methods:**

This analysis was based upon laboratory confirmed cases of salmonella gastroenteritis. Using data contained in CIDR, we examined completeness for certain non-mandatory fields (country of infection, date of onset of illness, organism, outcome, patient type, and ethnicity). We matched the CIDR data with the dataset provided by the national *Salmonella *reference laboratory (NSRL) to which all *Salmonella *spp. isolates are referred for definitive typing. We calculated the main median time intervals in the flow of events of the notification process.

**Results:**

In total, 416 laboratory confirmed *Salmonella *cases were captured by the national surveillance system and the NSRL and were included in the analysis. Completeness of non mandatory fields varied considerably. Organism was the most complete field (98.8%), ethnicity the least (11%). The median time interval between sample collection (first contact of the patient with the healthcare professional) to the first notification to the regional Department of Public Health (either a clinical or a laboratory notification) was 6 days (Interquartile 4-7 days). The median total identification time interval, time between sample collections to availability of serotyping and phage-typing results on the system was 25 days (Interquartile 19-32 days). Timeliness varied with respect to *Salmonella *species. Clinical notifications occurred more rapidly than laboratory notifications.

**Conclusions:**

Further feedback and education should be given to health care professionals to improve completeness of reporting of non-mandatory fields. The efficiency of reporting was similar to that published elsewhere. Delays in the reporting system at present mean that although the system is of value in facilitating comprehensive reporting it is unlikely it can be relied upon for rapid detection of outbreaks at an early stage. Direct person-to-person, communication between clinical and reference laboratories and public health practitioners remains a critical element of the surveillance system for rapid outbreak detection.

## Background

Salmonellosis is the second commonest cause of bacterial gastroenteritis in Ireland after *Campylobacter *spp [1]. In 2008, the Irish Health Protection Surveillance Centre (HPSC) received 449 *Salmonella *notifications that corresponded to a crude incidence rate of 10.8/100000. Included in these cases were 22 *Salmonella *outbreak notifications associated with 79 cases with an associated hospitalisation rate of 25%.

The aims of the national Irish *Salmonella *surveillance system are:

1. To monitor the incidence of salmonellosis and to assess the burden of illness in the Irish population

2. To detect any changes in predominant serovars/strains over the time

3. To detect clusters and to take appropriate control measures when outbreaks are identified

4. To describe outbreak transmission routes and monitor potential emerging reservoirs.

Published reports have shown that electronic reporting improves timeliness and/or completeness of data [2-6]. Electronic reporting of *Salmonella *cases was established in Ireland in 2004 and combines information generated and collated by the regional Departments of Public Health (DPH) in the eight Irish Health Service Executive (HSE) areas, the primary microbiological laboratories and the National *Salmonella *Reference Laboratory (NSRL). At a national level, the NSRL performs an important role in assisting in the surveillance of *Salmonella *enterica infection and understanding epidemiology by routine testing of an extended panel of antimicrobial agents, serotyping, phage typing and molecular analysis of submitted isolates. The HPSC is responsible for collation of data, detection and investigation with partner agencies of national outbreaks, provision of expert advice, operational support during outbreaks and incidents when requested and publication of a national annual report.

Best evidence suggests that evaluation of surveillance systems should be conducted on a regular basis to ensure that such systems meet their intended objectives [7-9]. A full evaluation of any surveillance system should encompass the following features: simplicity, flexibility, data quality, acceptability, sensitivity, predictive value positive, representativeness, timeliness and stability [8].

In this report, we aim to describe completeness of the data and the timeliness of case notification.

## Methods

### The Irish Surveillance System for Salmonella Notification

A national case definition for salmonellosis, based upon the standard European Centre for Disease Prevention and Control salmonella case definition is in use in Ireland. A case is defined as having a clinical picture compatible with salmonellosis e.g. diarrhoea, abdominal pain, nausea and sometimes vomiting. Cases may also be asymptomatic. A confirmed case is a clinically compatible case that is laboratory confirmed, while a probable case is a laboratory confirmed isolate without clinical information or a case with clinical symptoms that has an epidemiological link.

*Salmonella *infection is one of the 68 infections for which notification is mandatory in Ireland *(Infectious Diseases (Amendment) (No. 3) Regulations 2003, S.I. No. 707 of 2003)*. Cases are reported through a web-based Computerised Infectious Disease Reporting system (CIDR). CIDR was introduced in 2004 and was fully implemented in six out of eight HSE areas and in 16 out of 36 primary laboratories in hospitals (but none of the small number of private laboratories), and in the NSRL by 2008. A confirmed case should have a clinical and a laboratory notification. Probable cases reported on the basis of an epidemiological link will only have a clinical notification. As not all primary laboratories have live access to the CIDR system, laboratory notification data are entered on the CIDR system by either of two processes. The majority of cases are diagnosed and reported by primary laboratories where CIDR is in use. For these cases, laboratory notifications are recorded directly on CIDR by laboratory personnel. For the minority of cases notified by primary laboratories where CIDR is not already in use, laboratory notifications are forwarded to the DPH where they are entered on CIDR. Clinicians are informed of positive diagnostic findings by laboratory personnel, at which point, clinicians should notify confirmed *Salmonella *cases. Clinical notifications consist of a standardized form filled out by the practitioner which is registered on CIDR by staff in the DPH in each HSE area. To ensure that cases are not reported in duplicate on the system, clinical and laboratory notifications corresponding to same case are matched at the DPH level using name, date of birth and address information.

In Ireland, it is standard practice that human clinical *Salmonella *isolates are referred to the NSRL for definitive typing (serotyping +/- phage typing). Where the primary laboratory is live on CIDR, this enhanced microbiological information is uploaded directly on CIDR by NSRL staff, where it is forwarded to the DPH in a two stage process via the primary laboratory. Where the primary laboratory is not yet live on CIDR, this information is reported to the DPH by other channels. To ensure a high level of completeness for the variable 'serotype' on CIDR, the serotype details for isolates from laboratories where CIDR is not already in use are updated manually on CIDR by the DPH. NSRL independently maintains a laboratory database on all isolates received, an extract of which is sent monthly to the HPSC, for data validation purposes.

At the HPSC level, all data entered into CIDR are anonymised and transferred to an SQL database which is the master data matrix containing all national notification data. Although not all participants in the surveillance system have direct access yet to CIDR, the salmonella surveillance system on CIDR covers the entire Irish population by receiving data directly, or indirectly through the DPH, from clinicians and all of the publicly and privately owned diagnostic laboratories.

### Inclusion/exclusion criteria

All CIDR notifications of confirmed cases in 2008, where a matching record on the NSRL laboratory database could be identified, were considered eligible for inclusion in the study. As HPSC personnel do not have access to named patient data, matching was performed on the basis of gender, date of birth, HSE-area, serotype, and/or where the DPH has already indicated a match. Additional variables from the NRSL dataset were merged with the CIDR variables based on this matching.

### Evaluation methods

#### 1. Completeness

The following non mandatory fields were examined for completeness: country of infection, date of onset of illness, organism, outcome, patient type and ethnicity.

#### 2. Timeliness

Time intervals (the number of days (d) between key dates) were calculated for each case when the dates were available. Microsoft Excel™ was used for calculations. Once all intervals were calculated, inaccurate dates (primarily recognised by identifying negative time intervals) were validated and excluded. Stata V0.8 software (Stata Corporation) was used to calculate each median time interval and their 1^st ^(25%) and the 3^rd ^(75%) interquartiles (IQ).

### Examined time intervals (Figure 1)

**Figure 1:**
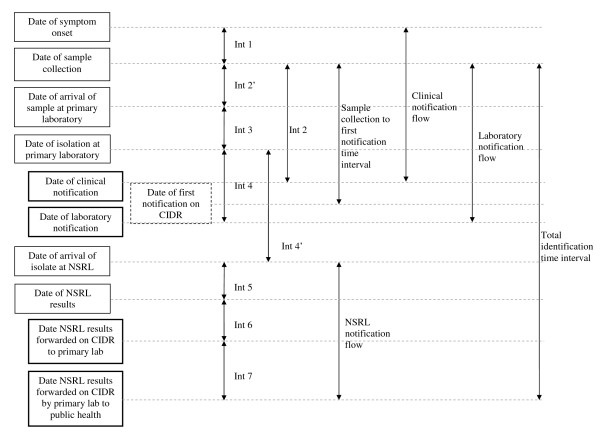
**Time intervals in the reporting of confirmed *Salmonella *cases, Ireland, 2008**. Int, interval; CIDR, Computerised Infectious Disease Reporting system; NSRL, National *Salmonella *Reference Laboratory. Bolded boxes indicate time points of data entry to CIDR; hatches box indicates a virtual date - earliest of clinical vs laboratory notification.

The following main time intervals were identified and studied in detail.

1. The time between sample collection (which was considered as the time of patient's first visit to the clinician) and the first notification date on CIDR (this could be either a laboratory or a clinical notification) was the time interval chosen to assess the overall performance of the system. It corresponds to the time between the patient coming into contact with the health care services and a proxy measure for the public health authorities becoming aware of the case. This was named the "*sample collection - first notification time interval". *We then examined each time interval in the flow of events in the clinical and laboratory notifications respectively.

2. The *clinical notification flow *is the sequence from the date of onset of symptoms to the date of clinical notification illustrated as interval 1 (int1) and interval 2 (int2).

3. The *laboratory notification flow *is the sequence between the date of specimen collection and the date of primary laboratory notification illustrated as interval 2' (int2'), interval 3 (int3) and interval 4 (int4).

4. The *NSRL notification flow *is the time sequence between the date the specimen was received at NSRL and the date when NSRL serotyping +/- phage typing results are forwarded to the DPH on CIDR via the primary laboratory (using the CIDR system). It is illustrated as interval 4' (int4') to interval 7 (int7).

5. To assess notification practice in term of availability of definitive *Salmonella *characterisation (serotyping +/- phage typing) on the system, the time interval between date of sample collection and forwarding of the serotyping +/- phage typing results by the primary laboratory to the DPH was examined. It is named the *total identification time interval *and illustrated as "TOTAL" in figure 1.

6. A specific analysis of the time interval to complete analysis of serotyping/phage typing at NSRL illustrated as interval 5 was made for the three *Salmonella *serotypes most commonly identified in 2008, namely *Salmonella *Enteritidis, *Salmonella *Typhimurium and *Salmonella *Agona.

7. With respect to those cases diagnosed in primary laboratories that are live on the system, a comparison of length of interval between each stream was undertaken.

## Results

A total of 449 cases were recorded onto CIDR but it was not possible to identify matching isolates in 33 (7%) cases in the NSRL database. Of the 433 *Salmonella *isolates recorded in the NSRL database, no CIDR notifications could be identified for 17 of them (4%). In total, 416 cases had both a CIDR record and a matching record in the NSRL database (92.7% of CIDR notifications). Of these, 294 (71%) had a laboratory notification from a primary laboratory live on the system. These 416 cases formed the basis for the subsequent analysis.

### Completeness for non mandatory fields in CIDR

Completeness varied for non mandatory fields (Table 1). The organism field was very complete (98.8%). Patient type and outcome fields were relatively incomplete: 62.9% and 33.9%. Almost two third of cases had a date for onset of symptoms specified (62.5%). Country of infection field was complete in about half of records (53.6%). Information on ethnicity was available for only 45 records (11%).

**Table 1:** Completeness of non mandatory fields in *Salmonella *notifications, Ireland, 2008 (N = 416)

Field	Number of reports	Percentage of total reports (%)
Organism	411	98.8
Patient type	262	62.9
Onset symptoms	260	62.5
Country of infection	223	53.6
Outcome	141	33.9
Ethnicity	45	11.0

### The timeliness.(table 2)

**Table 2:** Value of the examined time intervals, Salmonella notification, Ireland, 2008

Interval description	n	Median (IQ)
Sample collection - first notification interval	167	6 (4 - 7)
Interval 1: Onset of symptoms - sample collection	130	4 (0 - 6)
Interval 2: Sample collection - clinical notification	167	6 (5 - 8)
Interval 2': Sample collection - arrival of sample at primary laboratory	169	0 (0 - 1)
Interval 3: Arrival of sample at primary laboratory - Isolation at primary laboratory	197	3 (2 - 5)
Interval 4: Isolation at primary laboratory - laboratory notification	232	4 (1 - 8)
Interval 4': Isolation at primary laboratory - arrival of sample at NSRL^b^	398	4 (3 - 6)
Interval 5: Arrival of sample at NSRL^b ^- serotyping/phage typing results	416	5 (3 - 7)
Interval 6: Forward of serotyping/phage typing results by NSRL to primary laboratory	234	9.5 (6 - 18)
Interval 7: Forward of serotyping/phage typing results by primary laboratory to the DPH^c^	234	4 (1 - 7)
Total identification time interval : Sample collection - NSRL results uploaded on CIDR	154	25 (19 - 32)

^a^25%-75% Interquartile interval (IQ)

^b^National *Salmonella *Reference Laboratory

^c^Department of Public Health

#### The sample collection - first notification time interval

**Figure 2:**
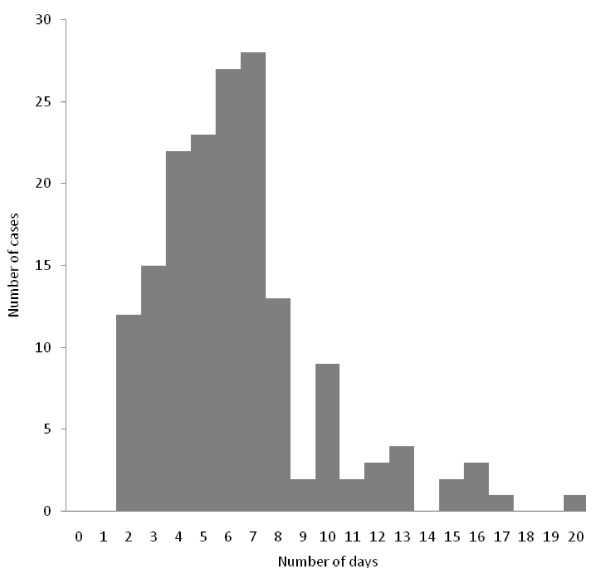
Distribution of the sample collection-first notification interval into CIDR*, Ireland, 2008 (n = 167). CIDR, Computerised Infectious Disease Reporting system. This interval represents the time between the patient coming into contact with the health care services and the public health authorities becoming aware of the case.

This time interval was calculated for 167 records (40.1%). Its distribution was skewed to the right (Figure 2). The median sample collection - first notification time interval was 6d (IQ 4-7d).

#### Median time intervals in the clinical notification flow

This describes the time interval between onset of symptoms and clinical notifications on CIDR. The median time interval between onset of symptoms and date of sample collection (Int1, n = 130) was 4d (IQ 0-6d). After sample collection it took a median time interval of 6d (IQ 5-8d) before clinical notification of the case (Int2, n = 167).

#### Median time intervals in the primary laboratory notifications flow

Most of the samples collected arrived at the primary laboratory on the day of collection giving a median time interval between date of sample collection and date of sample received (Int2', n = 169) of 0d (IQ 0-1d). Then the median time interval between the date the sample was received and the strain was isolated at the primary laboratory (Int3, n = 197) was 3d (IQ 2-5d). An additional median time interval of 4d (IQ 1-8d) was required to notify the primary laboratory results (Int4, n = 232) to the DPH.

#### Median time intervals in the reference laboratory notification flow

The median time interval between the isolation date at the primary laboratory and the date the isolate (Int4', n = 398) was received at the NSRL was 4d (IQ 3-6d).

The median time interval between receipt at the reference laboratory and serotyping/phage typing results (Int5, n = 416) was 5d (IQ 3-7d). The median time interval between the results date and the date the results were forwarded by NSRL to the primary laboratory via CIDR (Int6, n = 234) was 9.5d (IQ 6-18d). There was an additional median time interval of 4d (IQ 1-7d) to forward the results to the DPH by the primary laboratory via CIDR (Int7, n = 234).

#### The total identification time interval

The median total identification time interval, interval between the date of specimen collection and availability of serotyping +/- phage typing on CIDR (n = 154) was 25d (IQ 19-32d).

#### Timeliness by organism

A total of 65 different serotypes were identified by the NSRL during the study period. The most common serotypes identified were *Salmonella *Typhimurium (132 cases, 31%), *Salmonella *Enteritidis (121 cases, 29%) and *Salmonella *Agona (13 cases, 3.1%). The median total time interval for full identification at the NSRL was 6d for *Salmonella *Typhimurium and *Salmonella *Enteritidis (IQ 3-7d) and 3d for *Salmonella *Agona (IQ 2-4d). This reflects that additional time required for phage typing of *Salmonella *Typhimurium and *Salmonella *Enteritidis. Phage typing of *Salmonella *Agona is not performed.

In 2008, 11 of the 13 cases of *Salmonella *Agona notified formed part of a large European outbreak. The median time interval between first notification and onset of symptoms was 7d [IQ 5-8d]. The median time interval between onset of symptoms and specimen received to the reference lab was 8d [IQ 4-9d].

#### Comparison of the timeliness between laboratory and clinical notifications

For cases identified by primary laboratories which were live on the system (n = 294), it was possible to make a comparison between the timeliness of clinical and primary laboratory notifications (table 3). For 40% of cases, both notifications were received on the same day. One quarter of cases (25%) were notified in the first instance by the primary laboratory; the median time interval between that notification and the subsequent clinical notification was 1d (IQ 1-2d). One third of cases (35%) were notified in the first instance clinically; the median time interval between that notification and the subsequent laboratory notifications was 6d (IQ 3-12.5d). This suggests that clinicians were more prompt in notifying than laboratories.

**Table 3:** Timeliness of primary laboratory notifications and clinical *Salmonella *notifications, Ireland, 2008 (N = 294)a

Notification way	Percentage of total notification (%)	Median	**IQ**^**d**^	Mean	**SD**^**e**^
Laboratory/Clinical^b^	26	1	01/02/10	1.8	1.3
Clinical/Laboratory^c^	35	6	3 - 12.5	9.9	12.4

^a^Only cases identified by primary laboratories which were live on the system are included

^b^Clinical notifications (after arrival of laboratory notifications)

^c^Laboratory notifications (after arrival of clinical notifications)

^d^25%-75% Interquartile interval (IQ)

^e^Standart Deviation (SD)

## Discussion

This is the first study which assesses the completeness and the timeliness of the *Salmonella *notifications in Ireland since the implementation of the new national Computerized Infectious Disease Reporting (CIDR) in 2004. *Salmonella *was chosen as it remains a major bacterial cause of gastroenteritis in the Irish population and the surveillance system is well developed and stable over many years. The occurrence of regular outbreaks of salmonellosis (in 2008 an extended foodborne *Salmonella *Agona outbreak originating in Ireland resulted in 163 cases in Ireland, the UK and Europe) underlines the necessity of timely case reporting at a national level to detect cases and to describe the extent of outbreaks (national and regional) in order to apply rapid and effective control measures. Additionally, the emergence of unusual or locally uncommon strains of *Salmonella *due to an increasing numbers of exposures to new reservoirs [10] underlines the necessity for monitoring this disease with precise and timely clinical and microbiological data.

In our study, we were able to identify 416 out of 449 (92.7%) Irish salmonellosis cases that had both a CIDR notification and an NSRL record. For those 33 records on CIDR for which we could not identify a corresponding record in the NSRL dataset, possible likely explanations included that the isolate was never sent to the NSRL or that we were unable to match between the two datasets based on the identifiers available to us. For evaluation of completeness, we chose six variables which were non mandatory from a notification perspective but that described important elements of *Salmonella *epidemiology. Completeness of these six fields varied. This meant that important measures of the epidemiology of *Salmonella *and the responsiveness and sensitivity of the surveillance system (particularly country of infection, onset symptoms, outcome and patient type - hospital admission - a proxy for disease severity) may be inadequate. The variable *Country of infection *has the potential to be immensely valuable in allowing us to distinguish between indigenous and imported cases of *Salmonella *Enteritidis and to evaluate true trends in indigenous cases. A better knowledge of trends in indigenous cases allows a better assessment of control measures taken in respect of the *Salmonella *reservoirs in Ireland. For example, the Irish Department of Agriculture, Fisheries and Food operates a *Salmonella *control programme in poultry, which are known reservoirs of *Salmonella *Enteritidis. Reviewing the trends in serotype distribution of indigenous cases will permit us to comment on the effectiveness of such programmes at preventing cases of *Salmonella *Enteritidis associated with food consumed in Ireland. Ireland has become a multicultural and multiethnic society and the parameter *Ethnicity *has the potential to provide important pointers in the case of food contamination (either at source or in a food outlet) associated with particular ethnic groups. That only about 10% of cases have this information completed means that a potentially important source of information is lost. Completion of *Patient type and Outcome *fields are important proxy measures of disease severity. Monitoring severity of disease is important, especially if new vehicles of infection or serotypes emerge producing altered clinical patterns.

The notification system for *Salmonella *is complex. Altogether, twelve time intervals were identified in the complete notification process which included clinical and primary laboratory notifications as well as integration on CIDR of results of serotyping and phage typing. The main purpose of determining the timeliness of these intervals was to assess the rate limiting steps in the surveillance of salmonellosis on CIDR, as timely access to serovar and phage type information is important to rapidly identify and manage outbreaks. However, for those primary laboratories which were not live on CIDR (20 out of 36 which corresponds to 122 out of 416 notifications), timeliness of laboratory notifications onto CIDR could not be assessed.

The time interval between a primary laboratory identifying an isolate as being *Salmonella *and the first reporting of this case to the DPH is short (4d). This time interval was similar to that reported by Sweden [11]. In terms of public health action (especially outbreak identification or exclusion of high risk cases) there is a need for the diagnosing clinician to report *Salmonella *cases in a timely manner. The shorter the delay in reporting, the greater will be the opportunity for effective public health action.

Timely access to serotype and phage type information by PH is important to assist early detection of clusters and outbreaks. Referral of isolates from primary laboratories to the NSRL took a median time of 4d, as this included the time required to package and transport isolates from the primary laboratory to the NSRL. The full identification at the NSRL took a median time of 5d permitting identification of clusters in a timely way by NSRL personnel which are communicated promptly to PH using informal channels (telephone, emails). In our evaluation, the longest delays identified were the formal reporting of the final typing result by the NSRL to the primary laboratory (interval 6, 9.5d (IQ 6-18)) via CIDR and by the primary laboratory to the DPH via CIDR (interval 7,4d (IQ 1-7)). The formal process for reviewing typing information through CIDR from the NSRL is not at present sufficiently timely for outbreak detection. However the process remains a valuable tool to monitor the incidence of the disease and to describe its main epidemiological characteristics. A proposed new laboratory information system at the NSRL will permit automated upload of typing information into CIDR, thus reducing the time required for this step.

This full process has a median time of 25d which is equal to the delay reported *by South *Wales [12] but longer than the time required in South Australia (14d) [13] and in King County Washington (16d) [14]. However, according to the descriptions given in these reports, important differences may have contributed to shorter reporting times. For example, in King County Washin*gton, phag*e typing was not part of the identification process. Some limitations apply to our results. The one year study period was short. However, it was selected as many public health areas and laboratories were live on CIDR by that time. For those laboratories not live on the system, comparisons of the timeliness of clinical and laboratory notifications could not be made. Moreover, we did not remove weekend days from the interval calculation. The justification for removing weekend days is that routine work is generally not performed on these days, and this would have improved the apparent timeliness of results. However we consider that it appropriate to include weekend days as all days are equally relevant to the spread of infection. The intervals calculated may not be representative of the true delay in turnaround as many dates were not available. This study provided baseline data on timeliness that will allow subsequent comparison once all regions are live on CIDR.

## Conclusions

Efforts should be made to continue to improve the completeness of non-mandatory variables and to improve the timeliness of reporting (especially definitive typing data) through CIDR. For local clusters of *Salmonella spp*., CIDR appears timely enough for identification and action by local public health personnel. For more diffuse outbreaks which rely on detailed typing information, local and national public health agencies should continue to rely on timely alerts received via non-CIDR channels (i.e. telephone/email) for cluster identification.

## Competing interests

The authors declare that they have no competing interests.

## Authors' contributions

PMK and PG initiated the project. NN wrote the study protocol, designed the analytic plan, performed the statistical analysis and wrote up the manuscript. Data from the National *Salmonella *Reference Laboratory were validated and collated by ND. PG assisted in the interpretation of the final results and collaborated to the writing of this manuscript. PMK helped in interpreting the final results and provided critical feedback. ND and MC reviewed the final paper and provided critical feedback. All authors read and approved the final manuscript.

## Pre-publication history

The pre-publication history for this paper can be accessed here:

http://www.biomedcentral.com/1471-2458/10/568/prepub
